# Micronutrient-rich dietary intake is associated with a reduction in the effects of particulate matter on blood pressure among electronic waste recyclers at Agbogbloshie, Ghana

**DOI:** 10.1186/s12889-020-09173-8

**Published:** 2020-07-06

**Authors:** Sylvia A. Takyi, Niladri Basu, John Arko-Mensah, Paul Botwe, Afua Asabea Amoabeng Nti, Lawrencia Kwarteng, Augustine Acquah, Prudence Tettey, Duah Dwomoh, Stuart Batterman, Thomas Robins, Julius N. Fobil

**Affiliations:** 1 Department of Biological, Environmental & Occupational Health Sciences, School of Public Health, University of Ghana, P. O. Box LG13, Legon, Accra, Ghana; 2 Faculty of Agricultural and Environmental Sciences, McGill University, Montreal, Canada; 3 Department of Biostatistics, School of Public Health, University of Ghana, Accra, Ghana; 4 Environmental Health Sciences, School of Public Health, University of Michigan, Ann Arbor, MI USA

**Keywords:** Diastolic blood pressure, E-waste recyclers, Heart rate, Particulate matter, Pulse pressure, Micronutrients, Systolic blood pressure

## Abstract

**Background:**

Informal recycling of electronic waste (e-waste) releases particulate matter (PM) into the ambient air. Human exposure to PM has been reported to induce adverse effects on cardiovascular health. However, the impact of PM on the cardiovascular health of e-waste recyclers in Ghana has not been studied. Although intake of micronutrient-rich diet is known to modify these PM-induced adverse health effects, no data are available on the relationship between micronutrient status of e-waste recyclers and the reported high-level exposure to PM. We therefore investigated whether the intake of micronutrient-rich diets ameliorates the adverse effects of ambient exposure to PM_2.5_ on blood pressure (BP).

**Methods:**

This study was conducted among e-waste and non-e-waste recyclers from March 2017 to October 2018. Dietary micronutrient (Fe, Ca, Mg, Se, Zn, and Cu) intake was assessed using a 2-day 24-h recall. Breathing zone PM_2.5_ was measured with a real-time monitor. Cardiovascular indices such as systolic BP (SBP), diastolic BP (DBP), and pulse pressure (PP) were measured using a sphygmomanometer. Ordinary least-squares regression models were used to estimate the joint effects of ambient exposure to PM_2.5_ and dietary micronutrient intake on cardiovascular health outcomes.

**Results:**

Fe was consumed in adequate quantities, while Ca, Se, Zn, Mg, and Cu were inadequately consumed among e-waste and non-e-waste recyclers. Dietary Ca, and Fe intake was associated with reduced SBP and PP of e-waste recyclers. Although PM_2.5_ levels were higher in e-waste recyclers, exposures in the control group also exceeded the WHO 24-h guideline value (25 μg/m^3^). Exposure to 1 μg/m^3^ of PM_2.5_ was associated with an increased heart rate (HR) among e-waste recyclers. Dietary Fe intake was associated with a reduction in systolic blood pressure levels of e-waste recyclers after PM exposure.

**Conclusions:**

Consistent adequate dietary Fe intake was associated with reduced effects of PM_2.5_ on SBP of e-waste recyclers overtime. Nonetheless, given that all other micronutrients are necessary in ameliorating the adverse effects of PM on cardiovascular health, nutrition-related policy dialogues are required. Such initiatives would help educate informal e-waste recyclers and the general population on specific nutrients of concern and their impact on the exposure to ambient air pollutants.

## Background

Ambient air pollution remains an environmental health problem, especially in low- and middle-income countries (LMICs). In the year 2016, ambient air pollution was responsible for 4.2 million deaths and caused 17% of ischemic heart disease and stroke [1]. Specifically, in Ghana, it is estimated that 17,000 people die yearly from air pollution-related causes [2]. The informal-level recycling of electronic waste (e-waste), largely using crude methods, is known to release pollutants; mainly, particulate matter (PM) into the ambient air. Other components of the pollutants include nitrogen dioxide (NO_2_), sulfur dioxide (SO_2_), carbon monoxide (CO), heavy metals, rare earth metals and persistent organic compounds such as polychlorinated biphenyls (PCBs). Such environmental pollutants, when inhaled over time, present severe pulmonary and cardiovascular health threats [3–5]. For example, PM when inhaled moves through the pulmonary endothelium and enters the bloodstream [6] where it induces hypertension, airway irritation, coughing, difficulty breathing, reduced lung function, non-fatal heart attacks, atherosclerosis, irregular heartbeat, anemia and in extreme cases indirectly causes early death due to lung cancer [7, 8]**.** Additionally, the exposure to PM often induces systemic inflammation and oxidative stress, which contribute to the pathophysiology of several neurological and cardiovascular diseases [9–12]. Particulate matter of diameter ≤ 2.5 μm (PM_2.5_) in particular induces endothelial dysfunction characterized by impaired vasodilation, pro-inflammatory and prothrombotic responses [13, 14]. This may augment systemic vascular resistance, leading to the development of hypertension.

Emerging evidence indicates that adequate nutrition may reduce the harmful effects of most air pollutants [15–18]**.** Micronutrients-rich diets contain both antioxidant and anti-inflammatory properties, which may reduce the risk of vulnerability to oxidative stressors associated with exposure to particulate matter [15, 19–21]. Adequate dietary intake of calcium (Ca), zinc (Zn), and magnesium (Mg) have been suggested to enhance endothelial function and further improves vascular and circulatory efficiency [22–27]. Furthermore, micronutrients such as copper (Cu), selenium (Se), zinc (Zn), and vitamins (A, C, and E) serve as antioxidants that influence the body’s defenses against PM_2.5_ exposure. These antioxidants terminate the chain reactions of reactive oxygen species (ROS) by removing free radical intermediates and also inhibit other oxidation reactions in order to reduce blood pressure (BP) [28, 29]. Micronutrients cannot be synthesized by the body and must, therefore, be acquired in sufficient quantities through food consumption to maintain normal physiological functions [30]. As a result, their deficiency in human nutrition remains a critical global health issue [31–33].

Generally, studies investigating the potential modifying effect of micronutrient-rich diet intake on air pollutant-associated hypertension are limited [30, 34–38]. Moreover, available studies have generally not considered informal sector workers who tend to be particularly vulnerable due to the nature of their work environment. Well-designed and robust studies are therefore needed to better understand how micronutrient-rich dietary intake can counteract the adverse effects of PM_2.5_ exposure on BP, especially when these diets contain both antioxidant and anti-inflammatory properties that may reduce the risk of vulnerability to ambient air pollutants [15, 19–21].

In Ghana, air pollution due to informal e-waste recycling as well as from other sources, e.g., bio-mass burning and traffic-related emissions, remain a public health concern. In the year 2016, for instance, the annual average PM_2.5_ concentration in the capital, Accra, was 55 μg/m^3^. This concentration is well above the World Health Organization (WHO)-recommended annual guideline of 10 μg/m^3^. Agbogbloshie, our study site, is situated in central Accra and provides a livelihood for many people. Prevailing work-related activities include informal e-waste recycling. Aside from the toxic exposures, informal e-waste recycling presents, the activity is physically demanding and thus may increase the requirement for nutrient intake from diet. This study addressed a critical knowledge gap regarding the population of e-waste recyclers by answering the following questions: (1) Do e-waste recyclers consume micronutrient-rich diets? (2) Is there a relationship between dietary micronutrient intake and BP? (3) Does dietary micronutrient intake modify the effect of PM_2.5_ on BP among e-waste recyclers at Agbogbloshie and Madina-Zongo controls (non-e-waste recyclers)? This study estimated PM exposures in the breathing zone of informal e-waste recyclers at Agbogbloshie relative to non-e-waste workers at Madina Zongo, the control site. We did not focus on source apportionment of PM, hence the proportion of PM exposures specific to e-waste disposal. Further, we did not collect data on alternative emissions such as vehicle emissions and biomass burning, so these alternate sources were not studied. The interest of this analysis was to investigate the effect of micronutrient-rich diets on the relationship between PM exposure (regardless of the source of PM) and blood pressure – to answer one of the several research questions of a larger GEO-Health II study. However, it is important to mention that the choice of the exposed population at Agbogbloshie informal e-waste dumpsite and the control population at Madina Zongo, a suburb located more than 10 km from Agbogbloshie was informed by the broader interest in understanding the effects of exposures due to informal e-waste recycling activities on respiratory morbidity and other human health outcomes.

## Methods

### Study design

The study data were drawn from the Geo-Health-II longitudinal cohort study, as described by Amoabeng Nti et al. [39], which principally focused on respiratory health outcomes. Briefly, the study was based on three data collection waves among e-waste recyclers and non-e-waste recyclers to achieve seasonal variation in work patterns and personal exposure; i.e., wave I [March–April 2017] (dry season), wave II [July–August, 2017] (rainy season), and wave III [March–April 2018] (dry season). As detailed in past studies at Agbogbloshie [40–43], a community durbar was organized to familiarize participants with the study’s objectives and procedures. We ultimately recruited 142 e-waste workers from Agbogbloshie and 65 non-e-waste recyclers (from Madina Zongo) into one or more study waves as detailed earlier [39]. For subsequent waves (II and III), participants were recalled through phone calls. Community representatives also helped in recalling previously recruited participants. These helped reduce participant loss to follow up.

The inclusion criteria for participants of Agbogbloshie included adult males aged 18 years and above and have worked at the e-waste site for at least 6 months. Similarly, non-e-waste recyclers had similar age, culture, and food consumption characteristics as e-waste recyclers. However, they had never worked at the e-waste site. Also, these non-e-waste recyclers must have lived at the Madina-Zongo for at least 6 months. Study participants were compensated with 50 Ghana cedis (approximately US$10, roughly an average day’s wage), lunch, and a T-shirt at each wave. The University of Ghana and the University of Michigan Institutional Review Boards (IRB) approved the study protocols. The local chief of Agbogbloshie and Madina-Zongo permitted and allowed the research team to conduct this study.

### Study site

Agbogbloshie is famously known for informal e-waste recycling and is located in central Accra. It is noteworthy that this e-waste site is situated on the banks of the Odaw river and the Korle-Lagoon, approximately covering an area of 1.46 km^2^ and has an estimated population of 80,000 people [44–46]. An informal community, popularly known as “Old Fadama” lies southwest of the recycling area. The community houses most of the recyclers and other informal operators such as traders and street hawkers. The vast majority of people working in the scrap metal yard are young men and boys, culturally Dagombas or Konkombas who migrated from the northern part of Ghana in search of greener pastures. Graphically, the recycling area is flat with closely-mounted small open sheds from which recyclers operate. This site receives and informally recycles a collection of obsolete electronic items such as fridges, television, mobile phones, computers, and cars. The informal recycling methods employed consist of open-air burning of wires to recover copper, as well as manual off-loading and dismantling of equipments/ devices.

**Fig. 1:**
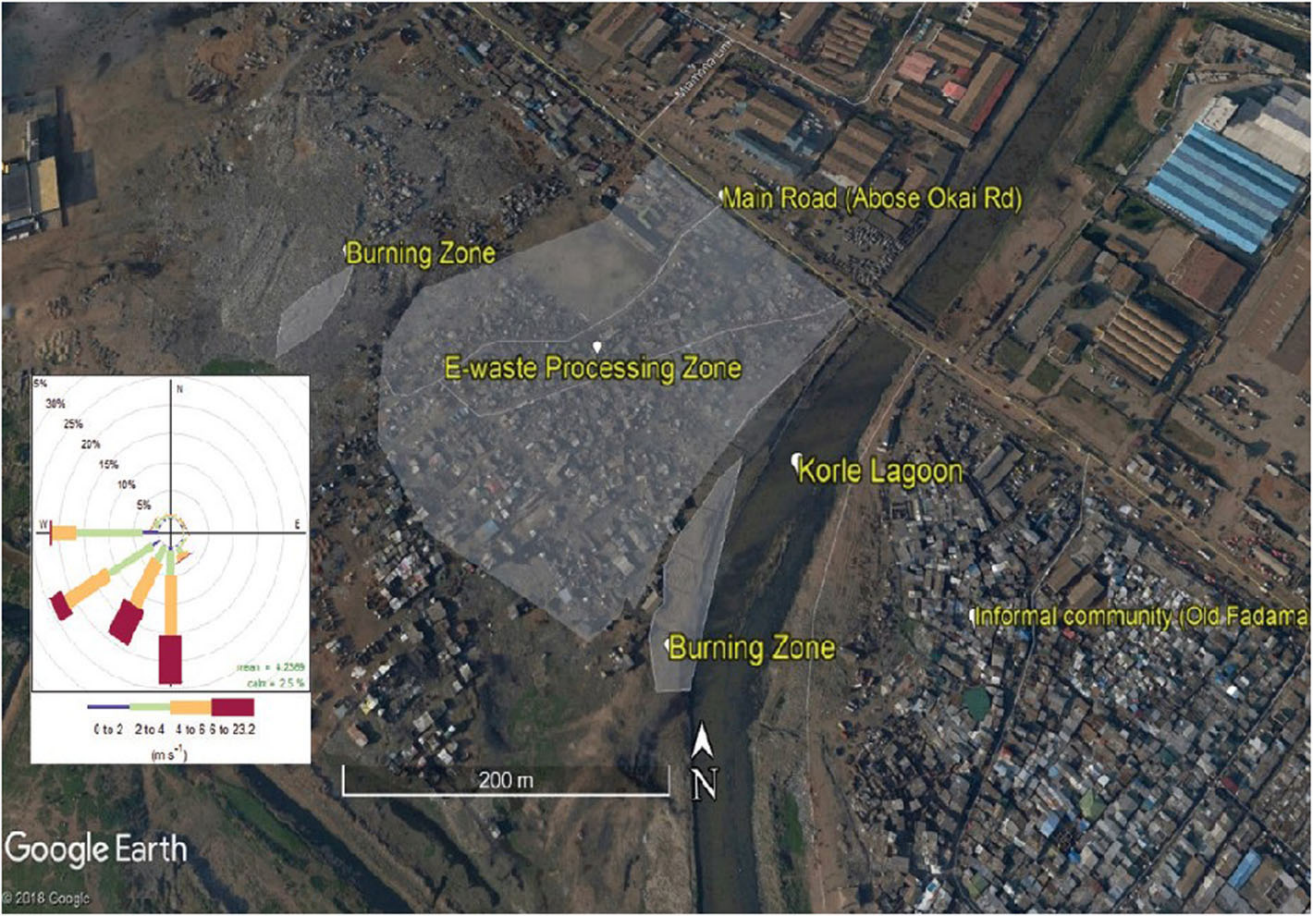
Map of Agbogbloshie electronic-waste recycling site. The area highlighted is the actual site where data were collected. The map was drawn using Google Earth Fig. 1: Map of Agbogbloshie electronic-waste recycling site. This site is located in Accra, Ghana. The large area marked grey is the e-waste processing zone where tasks such as dismantling, sorting, weighing, and burning and trading are carried out. To the south of the e-waste site is the Korle-Lagoon and the informal community called old Fadama. The map was drawn using Google Earth Pro V 7.3.2.5776. (10 July 2015).© Google, 2019. Source: Laskaris et al. [47]

**Fig. 2:**
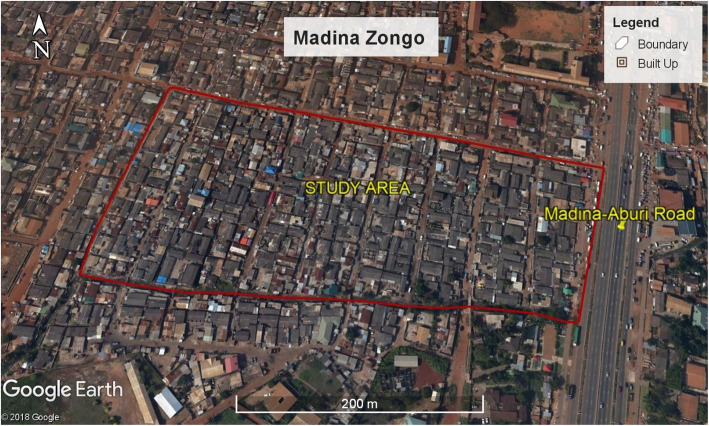
Map of Madina Zongo, located in Accra, Ghana. Pro V 7.3.2.5776. (10 July 2015).© Google, 2019

Apart from informal e-waste recycling, prevailing activities and businesses consist of buying and selling foodstuffs such as yams and onions. Furthermore, Agbogbloshie is characterized by an extensive overlap of industrial, commercial, and residential zones. Generally, the Agbogbloshie scrap yard is noted for heavy clouds of smoke from typical daily burning of e-waste materials such as copper. The geographical location of Agbogbloshie is shown in Fig. 1. We further provide a pictorial view of Madina-Zongo in Accra, Ghana, in Fig. 2.

#### Field data collection procedures

##### Anthropometric and blood pressure measurements

Height was measured and corrected to the nearest 0.1 cm using a Seca Stadiometer (Seca; Germany), with the participant standing upright on a flat surface without shoes, and the back of the heels and the occiput against the stadiometer [48–50]**.** Weight was also measured and recorded to the nearest 0.1 kg using a portable Seca Scale (Seca770; Hamburg, Germany). The same model standard calibrated balance was used at both study sites. Body mass index (BMI) of each participant was calculated by dividing the weight in kilograms (kg) by height in meters squared (m^2^). The body weight was also measured at each time point of data collection to assess whether or not there was a measurable change in body stores.

BP was measured by a professional nurse using a sphygmomanometer with a portable cuff device (Omron model HEM 711 AC, Omron Healthcare Inc., Lake Forest, IL). We used the BP measurement guidelines by the US National Health and Nutritional Examination Survey method [37]. Participant’s BP readings were taken on the left brachial arm after a minimum of 10 min rest in the seated position, with the arm at heart level [51]. The average of three readings was used as the outcome variable [52]. In addition, the pulse pressure (PP), an indicator of arterial stiffness, was calculated as the difference between the systolic BP (SBP) and the diastolic BP (DBP) [53]. The mean arterial pressure (AP) was computed as (SBP + 2*DBP) /3 [54].

### Nutrient intake assessment

Data collection during each time point took place over a period of one to 2 weeks. Daily nutritional intake of participants was collected using a semi-structured 2-day 24-h recall guide. We conducted the 24-h recall twice to estimate the day-to-day variability per individual due to the variety of foods consumed on different days. Trained dieticians were employed to collect nutrition data in order to maximize the consistency of the interview format across study sites and further minimize between-site methodological biases. Interviewers obtained written informed consent from participants before undertaking this nutrition survey. The interview was conducted in the native or chosen language of the participants: Dagbani, Hausa, Twi, or English, to better ensure that participants thoroughly understood the questions posed in the 2-day 24-h recall guide. Our interview consisted of foods and beverages (e.g., the amount, time, and types) consumed on one weekday and 1 day of a just past weekend (Saturday or Sunday). In wave I, a total of 142 e-waste recyclers and 65 non-e-waste recyclers were interviewed face-to-face. During the follow-up waves (II and III), 138 e-waste recyclers and 65 non-e-waste recyclers were interviewed face-to-face. However, six (6) e-waste recyclers who at the time of the follow up had traveled back to the Northern part of the country for their annual farming duties were interviewed via telephone. We also used graduated food models to quantify foods and beverages consumed by each participant.

### Measures of real-time personal PM_2.5_ levels

For each wave, optical and gravimetric breathing zone PM levels were measured for both the exposed group in Agbogbloshie and the control group at Madina Zongo. Near continuous minute by minute, real-time PM_1_, PM_2.5_, PM_4_, PM_10_, and total suspended particles (TSP) were measured with an optical counter (Aerocet 831, Met One Instruments, Inc., OR, USA) that sampled at 2.83 L/m. For quality control, PM_2.5_ concentrations were considered invalid when TSP exceeded 2000 μg/m^3^. Gravimetric measurements were done for only PM_2.5_ using a size-selective impact sampler with a pre-weighed 47 mm Teflon filter (2 μm spore size, SKC PA, USA) and a flow rate of 10 L/min. This flow rate was used to help ensure an optimal load onto the filter even with variable wind conditions [55, 56]. All the equipment was contained in a customized backpack with inlets in the breathing zone of the participants. The e-waste recyclers were usually present at the worksite from about 7 am to 6 pm. A detailed study of their time-activity budgets revealed that they worked 6 to 7 days per week at an average of 10 h per day [47]. Our instruments were calibrated to sample for 4 h, and usually between the period of 8 am and 2 pm. In wave III, the sampling duration was reduced to approximately 2 h due to the high levels of PM from the Harmattan winds. Further description of the sampling process is described in Laskaris et al. [47].

#### Data analysis

##### Nutrient analysis

The nutrient intake data was converted into grams using Ghanaian Food Composition Tables. Furthermore, nutrient analysis was conducted using the ESHA F Pro software® to estimate individual micronutrient intake. After the nutrient analysis, data obtained from the ESHA F Pro comprised of amounts of calcium (Ca), magnesium (Mg), iron (Fe), zinc (Zn), copper (Cu), and selenium (Se) consumed. Also, the mean probability of micronutrient adequacy was computed to estimate the percentage of participants who met the Recommended Daily Allowance (RDA) for adult males [57] over time.

##### Statistical analysis

Firstly, each wave was analyzed independently of each other. At each wave, t-test statistics were used to compare between-group differences (e-waste recycler group vs. control) in the mean distribution of systolic, diastolic, pulse, arterial pressure, and heart rate, PM and BMI measures. Secondly, the data from the three (3) waves were merged to form the longitudinal format, which was then used for further analysis.

##### Micronutrient intake by e-waste and non-e-waste recyclers

The study compared the differences in the proportion of e-waste and non-e-waste recyclers who met the RDA of micronutrients using the z-test at each wave. This comparison was made by dichotomizing each of the outcome measures based on the United States Department of Agriculture (USDA) guidelines [58, 59] for adults. The USDA definition outlines the threshold for micronutrient adequacy using data obtained from the reported dietary micronutrient intake of participants. Sensitivity analysis was also conducted by comparing the actual mean distribution of the micronutrient intake between e-waste and non-e-waste recyclers at each wave using the Welch t-test. The Welch’s t-test was used because the variances of the outcome measures were not equal between the e-waste recyclers and the non-e-waste recyclers. After this, the ordinary least squares regression model with random effects was used to assess the impacts of daily income accrued and physical demands on dietary micronutrient intake in e-waste recyclers and non-e-waste recyclers.

##### Relationship between micronutrient intake and blood pressure levels

The study also assessed the relationship between BP and micro-nutrient intake using a multiple linear regression model with a robust standard error that controls for confounders.

##### Micronutrient intake and its association with PM_2.5_ and blood pressure levels

The study assessed the normality of all continuous outcome measures using the Shapiro Francia test [60]. Non-normal outcome measures were log-transformed before conducting further statistical analyses. We further conducted Hausman’s test to inform the choice of the fixed and random-effects model. Based on the results of the Hausman’s test (*p* > 0.05), the random-effects model was finally used to assess the effect of micronutrient intake on BP controlling for PM. All statistical tests were conducted using Stata® version 15 (StataCorp, College Station, Texas, USA).

## Results

### Health characteristics of e-waste recyclers and non-e-waste recyclers

Participants’ mean age was 27.6 ± 0.4 years. Overall, the mean SBP of e-waste recyclers was 120 mm Hg (standard error of the mean: SE = 1.1 mm/Hg) and mean DBP was 72.2 mm Hg (SE = 0.9 mm/Hg). The mean PP was 48.7 mm Hg (SE = 1.1 mm/Hg) and mean heart rate (HR) was 73.7 mm Hg (SE = 1.0 mm/Hg) for the three waves. Our study overtime found that mean arterial pressure (AP), systolic BP (SBP) and diastolic BP (DBP) were consistently higher in non-e-waste recyclers than e-waste recyclers. The PM_2.5_ levels were significantly higher in e-waste recyclers, especially at waves I and II. However, the mean PM_2.5_ level of non-e-waste recyclers at wave III was 80.4 micrograms per cubic meter (SE = 5.6 μg/m^3^), which is about four times the WHO 24-h guideline value of 25 μg/m^3^. Similar to that observed among the e-waste recyclers, the non-e-waste recyclers also exceeded this guideline at all time points. Further comparison between the health characteristics of e-waste and non-e-waste recyclers over time were made accordingly (Appendix 1A). When BP outcomes were compared over time, the study found a significant decline in SBP and AP of e-waste recyclers (Table 1). Although the BMI of e-waste recyclers significantly increased over time, non-e-waste recyclers were found to have a higher BMI reading, especially at waves I and II. Averagely, the e-waste recyclers had approximately worked for about 10 years at the Agbogbloshie dumpsite.

**Table 1:** Health characteristics of e-waste recyclers and non-e-waste recyclers in Accra-Ghana

	E-waste Recyclers Mean ± SE	Non-E-waste Recyclers Mean ± SE	T-test	***P***-value
Blood pressure at Wave I				
Systolic Pressure	123.06 ± 1.03	128.57 ± 2.12	− 2.63	0.01
Diastolic Pressure	73.61 ± 0.83	76.46 ± 1.50	− 1.79	0.07
Pulse Pressure	49.46 ± 0.94	52.11 ± 1.24	− 1.63	0.11
Arterial Pressure	90.09 ± 0.79	93.83 ± 1.63	− 2.33	0.02
Heart rate	73.69 ± 0.97	74.38 ± 1.64	−0.38	0.70
Blood pressure at Wave II				
Systolic Pressure	119.85 ± 1.03	127.00 ± 2.45	− 3.17	0.002
Diastolic Pressure	72.19 ± 0.82	75.52 ± 1.77	− 1.96	0.05
Pulse Pressure	47.67 ± 1.08	51.48 ± 1.53	− 2.00	0.04
Arterial Pressure	88.07 ± 0.74	92.68 ± 1.89	− 2.74	0.01
Heart rate	73.44 ± 1.14	69.36 ± 1.54	2.06	0.04
Blood pressure at Wave III				
Systolic Pressure	119.62 ± 1.38	129.13 ± 2.99	−3.31	0.001
Diastolic Pressure	70.90 ± 1.07	75.96 ± 1.96	− 2.43	0.01
Pulse Pressure	48.71 ± 1.19	53.18 ± 1.62	−2.12	0.03
Arterial Pressure	87.14 ± 1.04	93.68 ± 2.23	−3.03	0.003
Heart rate	73.82 ± 1.05	73.62 ± 1.93	0.10	0.92
Ambient exposure (μg/m^3^)				
PM_2.5_ (at Wave I)	80.39 ± 5.59	39.03 ± 2.34	4.95	< 0.001
PM_2.5_ (Wave II)	70.49 ± 2.45	49.50 ± 7.23	3.43	< 0.001
PM_2.5_ (Wave III)	70.49 ± 3.79	87.50 ± 14.56	−1.48	0.14
BMI				
Wave I	22.52 ± 0.25	23.82 ± 0.42	−2.83	0.01
Wave II	23.96 ± 0.30	24.14 ± 0.51	− 0.32	0.76
Wave III	24.18 ± 0.34	24.11 ± 0.59	0.11	0.92

*BMI* body mass index, *PM*_*2.5*_ particulate matter < 2.5 μm in diameter in μg/m^3^

### Estimates of dietary micronutrient intake and adequacy among e-waste and non-e-waste recyclers

A comparison of reported dietary intake of micronutrients between e-waste recyclers and non-e-waste recyclers is shown in Table 2 below. Mean micronutrient intake of Fe, Mg and Zn from the diet consumed were significantly different between e-waste and non-e-waste recyclers at wave I; dietary Fe (t (1) =2.70, *p* = *0.004*) and Zn (t (1) =2.81, *p = 0.01*) intake were significantly higher in e-waste than non-e-waste recyclers. In contrast, Mg intake was significantly higher in non-e-waste recyclers (*p < 0.05*). Also, nearly all e-waste and non-e-waste recyclers consumed adequate amounts of Fe from diet per the RDA at all waves analyzed. Furthermore, we compared dietary micronutrients of e-waste and non-e-waste recyclers overtime (Appendix 1E). To a large extent, micronutrients such as Ca, Cu, Se, and Mg were inadequately consumed in both study groups per the RDA guidelines (Table 2). We further assessed the effects of e-waste exposure, job task, and daily income earned on dietary micronutrient intake (Appendix 1B). Dietary Ca, and Fe intake were positively related to daily income of more than GH¢200 (~ 36 USD). Compared to participants who earned a daily earning of GH¢20, Zn intake was significantly related to all higher levels of daily income earned. Between recycler types, collectors significantly consumed higher amounts of Se than burners, dismantlers, and sorters.

**Table 2:** Dietary Micronutrient intake and adequacy of e-waste and non-e-waste recyclers at different time points

Micronutrient (mg)	RDA (mg)	Dietary Micronutrient Intake				% Micronutrients adequacy (=% > RDA)		
		E-waste Recycler	Non-E-waste recyclers			E-waste Recyclers	Non-E-waste recyclers	
		Mean ± SD	Mean ± SD	T-test	*P*-value	n (%)	n (%)	*p*-value
**WAVE I**								
Ca	1000	518.15 ± 318.26	521.68 ± 345.92	−0.07	0.95	7 (4.4)	2 (3.2)	0.57
Fe	8	26.35 ± 12.44	21.57 ± 9.85	2.70	0.004	127 (96.9)	62 (98.4)	0.18
Cu	2	1.10 ± 0.58	1.05 ± 0.63	0.55	0.59	13 (9.2)	3 (4.8)	0.28
Se	55	25.76 ± 18.74	34.87 ± 40.78	−2.16	0.10	22 (16.8)	15 (23.8)	0.24
Mg	400	46.98 ± 44.27	78.84 ± 63.13	−4.12	0.001	4(2.8)	0(0)	0.55
Zn	11	11.17 ± 4.06	9.38 ± 0.57	2.81	0.01	67(47.2)	18(28.6)	0.01
**WAVE II**								
Ca	1000	536.68 ± 273.46	560.80 ± 393.93	− 0.39	0.70	7(6.6)	3(6.0)	0.89
Fe	8	29.10 ± 12.29	23.65 ± 10.54	2.86	0.005	111(98.2)	51(100)	0.34
Cu	2	1.10 ± 0.54	0.98 ± 0.57	1.26	0.21	7(6.6)	3(6.0)	0.89
Se	55	32.26 ± 22.35	35.25 ± 26.98	− 0.66	0.51	19(16.8)	13(25.5)	0.19
Mg	400	45.99 ± 33.15	48.98 ± 37.56	−0.48	0.63	0(0)	1(2)	0.14
Zn	11	11.57 ± 4.10	9.51 ± 3.89	3.02	0.003	59(55.7)	12(24.0)	< 0.001
**WAVE III**								
Ca	1000	539.50 ± 228.41	574.71 ± 296.07	−0.70	0.48	5(4.9)	3(6.8)	0.64
Fe	8	29.21 ± 11.28	22.49 ± 9.60	3.67	< 0.001	104(99.1)	43(97.7)	0.52
Cu	2	1.16 ± 0.64	0.99 ± 0.71	1.32	0.19	12(11.8)	4(9.1)	0.64
Se	55	37.53 ± 24.94	40.05 ± 25.86	−0.51	0.61	28(26.7)	13(29.6)	0.72
Mg	400	41.58 ± 44.62	50.28 ± 40.66	−1.12	0.27	7(6.9)	2(4.6)	0.59
Zn	11	11.33 ± 3.84	9.80 ± 4.73	1.89	0.06	56(54.9)	13(29.6)	0.01

*Abbreviations*: *Ca* Calcium, *Fe* Iron, *Cu* Copper, *Se* Selenium, *Mg* Magnesium, *Zn* Zinc, *RDA* Recommended Daily Allowance

### Relationship between dietary micronutrient intake and BP

The adjusted models, as compared to the unadjusted models, showed only occasional and relatively small changes in associations between dietary micronutrient intake and measures of BP. In our unadjusted model, a significant inverse relationship was observed between Zn and SBP (β = − 0.03; 95% CI = − 0.05, 0.01, *p* = 0.02; Table 3) but not DBP (β = − 0.02; 95%CI: − 0.05, 0.01, *p = 0.24*), PP (β = 0.046, 95%CI: − 0.094, 0.002, *p = 0.05*) and HR (β = 0.009; 95%CI; − 0.030, 0.048, *p = 0.66*). In addition, a unit increase in dietary Ca intake reduced SBP by 0.03 mmHg (95% CI: − 0.044, 0.003*, p = 0.022*) and further decreased PP by 0.05 mmHg (95% CI: − 0.09, 0.01, *p* = 0.021). Iron (Fe) intake from diet also significantly reduced SBP levels by 0.03 mmHg (95% CI: − 0.05, − 0.01; *P* = 0.002). These reductions by Fe were also observed for PP and AP levels in the model. Even though no significant difference was found, micronutrients such as Ca, Zn, Se, Fe, and Cu marginally reduced the DBP. However, when the model was adjusted for income, BMI, smoking status, marital status, total calories consumed, and dietary diversity scores, Ca reduced SBP, PP, and HR, while Fe reduced the SBP, PP, and AP levels (Table 3). Further analyses based on multivariable regression models were conducted to determine the effect of micronutrient-rich dietary intake on BP of e-waste recyclers. It was found that every 1 mg intake of Fe-rich diets significantly reduced SBP of e-waste recyclers by 0.03 mmHg (95%CI: − 0.063, 0.00004; *p < 0.05*).

**Table 3:** Relationship between dietary micronutrient intake (mg) and BP (mmHg)

Dietary micronutrients (mg)	Systolic blood pressure (SBP) β (95% CI)	Diastolic blood pressure (DBP) β (95% CI)	Pulse pressure (PP) β (95% CI)	Arterial pressure (AP) β (95% CI)	Heart rate (HR) β (95% CI)
**Unadjusted**					
Ca	− 0.024* [− 0.044, 0.003]	− 0.001 [− 0.027, 0.023]	− 0.049 * [− 0.091, − 0.007]	− 0.012 [− 0.033, 0.008]	− 0.014 [− 0.049, 0.019]
Mg	0.01 [− 0.01, 0.02]	0.013 [− 0.001, 0.027]	−0.004 [− 0.027, 0.019]	0.009 [− 0.003, 0.020]	0.010 [− 0.009, 0.029]
Se	0.01 [− 0.003, 0.019]	−0.001 [− 0.014, 0.013]	0.022 [− 0.0003, 0.0441]	0.003 [− 0.008, 0.014]	−0.007 [− 0.025, 0.011]
Fe	− 0.03** [− 0.05, − 0.01]	−0.013 [− 0.039, 0.013]	−0.058** [− 0.101, 0.015]	− 0.023* [− 0.044, − 0.001]	− 0.003 [− 0.039, 0.032]
Zn	− 0.03* [− 0.05, 0.01]	− 0.02 [− 0.05, 0.01]	−0.046 [− 0.094, 0.002]	−0.022 [− 0.005, 0.0002]	0.009 [− 0.030, 0.048]
Cu	0.002 [− 0.12, 0.02]	− 0.005 [− 0.023, 0.013]	0.012 [− 0.017, 0.041]	− 0.002 [− 0.016, 0.012]	0.005 [− 0.019, 0.029)
**Adjusted**					
Ca	− 0.027* [− 0.054, − 0.004]	− 0.001 [− 0.035, 0.034]	−0.057* [− 0.113, − 0.001]	−0.014 [− 0.041, 0.014]	−0.048* [− 0.095, − 0.001]
Mg	0.005 [− 0.007, 0.016]	0.012 [− 0.003, 0.027]	− 0.004 [− 0.028, 0.020]	0.008 [− 0.003, 0.020]	0.014 [− 0.006, 0.034]
Se	0.006 [− 0.005, 0.017]	− 0.003 [− 0.018, 0.011]	0.020 [− 0.003, 0.043]	0.001 [− 0.011, 0.012]	− 0.008 [− 0.001, 0.011]
Fe	− 0.045** [− 0.073, − 0.016]	− 0.024 [− 0.061, 0.014]	−0.069* [− 0.130, − 0.009]	−0.034* [− 0.064, − 0.004]	−0.046 [− 0.098, 0.006]
Zn	− 0.026 [− 0.061, 0.009]	−0.027 [− 0.072, 0.017]	−0.030 [− 0.103, 0.043]	−0.027 [− 0.063, 0.009]	− 0.023 [− 0.001, 0.010]
Cu	0.014 [− 0.003, 0.031]	− 0.001 [− 0.023, 0.021]	0.034 [− 0.001, 0.070]	0.005 [− 0.012, 0.024]	−0.001 [− 0.031, 0.029]

*P*-value notations: *p* < 0.05*; *p* < 0.01** Random effect adjustment was made for income, BMI, smoking status, marital status, total calories consumed, dietary diversity scores in the model

### Effects of dietary micronutrient intake on the association between PM_2.5_ and BP

Generally, higher PM_2.5_ exposure was associated with a significant increase in HR (β: 0.061; 95%CI: 0.007, 0.116; *p = 0.03*) of e-waste recyclers at Agbogbloshie after adjusting for age, BMI, smoking status, total calories consumed and dietary diversity scores (Appendix 1C). However, in our joint effect model, Fe reduced SBP by 0.04 mmHg (95% CI: − 0.074, − 0.012; *p* < 0.01) and AP by 0.04 mmHg (95%CI: − 0.068, − 0.004; *p < 0.05*) after PM_2.5_ exposure (Table 4). Furthermore, Mg slightly increased DBP by 0.02 mmHg (95%CI: 0.001, 0.032; *p < 0.05*) and HR by 0.02 mmHg (95%CI: 0.002, 0.047; *p = 0.02*) among both e-waste and non-e-waste recyclers. Nonetheless, dietary Cu intake also increased PP by 0.04 mmHg (95%CI: 0.006, 0.079; *p < 0.05*) when both e-waste recyclers and non-e-waste recyclers were included in the model. Particularly in e-waste recyclers, 1 mg of Fe consumed was associated with a 0.04 mmHg reduction of SBP levels (95%CI: − 0.073, − 0.004; *p = 0.02*; Appendix 1D). Further in the model, 1 mg intake of Cu was associated with a 0.04 mmHg increase in PP among e-waste recyclers (95%CI: 0.001, 0.088; *p = 0.04*)*.*

**Table 4:** Effects of dietary micronutrient intake on the association between PM_2.5_ and BP in both e-waste and non-e-waste recyclers

Variables	Systolic blood pressure (SBP)	Diastolic blood pressure (DBP)	Pulse pressure (PP)	Arterial pressure (AP)	Heart rate (HR)
	β [95% CI]	β [95% CI]	β [95% CI]	β [95% CI]	β [95% CI]
PM_2.5_	−0.033* [− 0.054, − 0.013]	−0.027* [− 0.053, − 0.002]	−0.045* [− 0.088, − 0.003]	−0.030* [− 0.051, − 0.009]	0.022 [− 0.014, 0.060]
Ca	−0.029 [− 0.066, 0.008]	−0.001 [− 0.036, 0.034]	−0.042 [− 0.101, 0.016]	−0.010 [− 0.039, 0.019]	−0.045 [− 0.095, 0.006]
PM_2.5_	− 0.032* [− 0.053, − 0.011]	−0.025 [− 0.051, 0.001]	−0.045* [− 0.089, − 0.002]	−0.028* [− 0.049, − 0.007]	0.029 [− 0.008, 0.065]
Mg	0.006 [− 0.006, 0.019]	0.016* [0.001, 0.032]	−0.007 [− 0.033, 0.019]	0.012 [− 0.001, 0.024]	0.024* [0.002, 0.047]
PM_2.5_	− 0.030* [− 0.051, − 0.010]	−0.026 [− 0.051, 0.0003]	−0.040 [− 0.083, 0.003]	−0.027* [− 0.048, − 0.007]	0.027 [− 0.010, 0.064]
Fe	−0.043* [− 0.074, − 0.012]	−0.029 [− 0.069, 0.010]	−0.061 [− 0.126, 0.005]	−0.036* [− 0.068, − 0.004]	−0.029 [− 0.086, 0.028]
PM_2.5_	− 0.029* [− 0.050, − 0.008]	−0.026 [− 0.053, 0.001]	−0.037 [− 0.082, 0.008]	−0.027* [− 0.049, − 0.005]	0.028 [− 0.011, 0.066]
Se	0.007 [− 0.005, 0.018]	−0.001 [− 0.016, 0.014]	0.019 [− 0.006, 0.043]	0.002 [− 0.009, 0.014]	−0.009 [− 0.030, 0.012]
PM_2.5_	− 0.033* [− 0.054, − 0.013]	−0.027* [− 0.053, − 0.002]	−0.045* [− 0.087, − 0.002]	−0.030* [− 0.051, − 0.009]	0.025 [− 0.012, 0.062]
Cu	0.017 [− 0.0001, 0.035]	−0.001 [− 0.023, 0.021]	0.043* [0.006, 0.079]	0.008 [− 0.010, 0.026]	−0.003 [− 0.034, 0.029]
PM_2.5_	− 0.031* [− 0.052, − 0.011]	−0.025 [− 0.051, 0.001]	−0.043 [− 0.086, 0.00004]	−0.027* [− 0.048, − 0.006]	0.026 [− 0.011, 0.064]
Zn	−0.029 [− 0.066, 0.008]	−0.040 [− 0.086, 0.007]	−0.017 [− 0.094, 0.060]	−0.035 [− 0.072, 0.003]	−0.015 [− 0.084, 0.053]

Random effect adjustment was made for age, income, BMI, smoking status, marital status, biomass exposure, total calories consumed, and dietary diversity scores in the model. *p* < 0.05*, for which reason their values were boldened

## Discussion

Several studies have reported the adverse effects of PM_2.5_ on BP outcomes [18, 37, 61, 62], with few focusing on how intake of micronutrient-rich diets may ameliorate these effects. To the best of our knowledge, this study is the first-ever to examine the role of micronutrient-rich dietary intake in reducing the harmful effects of PM among e-waste recyclers. The study found that the consumption of micronutrients, including Ca Se, Zn, Cu, and Mg, were below the recommended intakes. Furthermore, PM_2.5_ exposures were higher in e-waste recyclers compared to non-recyclers at the control site. However, the control site was equally highly polluted as concentrations measured exceeded the WHO 24-h air quality guideline value of 25 μg/m^3^. These high PM_2.5_ levels recorded in the control site may perhaps be due to emissions from car exhausts (owing to high vehicular traffic in that area), dust from untarred roads, and smoke from open burning of rubbish and biomass and other sources. Furthermore, we found, as expected, that PM_2.5_ levels increased in the harmattan season. Higher PM_2.5_ levels were found to be associated with increases in HR levels in e-waste recyclers. This association is similar to findings by Breitner et al. [63] and Xie et al. [64]. In contrast to our study, Cole-Hunter et al. [65] and Dong et al. [66] found a decrease in HR when PM_2.5_ levels increased. Possible reasons why our results may differ from Cole-Hunter et al. [65] and Dong et al. [66] may include; geographic and temporal variability of PM_2.5_ sources and constituents between the different study sites as well as existing differences in sociodemographic characteristics such as age. Generally, BP in non-e-waste recyclers was significantly higher than in e-waste recyclers over time (*p < 0.05*). This is surprising because it was expected that e-waste recyclers (they are exposed to higher PM_2.5_ levels) would have higher BPs than the non-e-waste recyclers. The observed higher BP among the control group compared to e-waste recyclers may probably be due to their sedentary lifestyle [67, 68].

### Estimates of dietary micronutrient intake and adequacy among e-waste and non-e-waste recyclers

Dietary Fe was adequately consumed among e-waste recyclers and non-e-waste recyclers, perhaps most likely owing to their frequent intake of traditional green leafy soups. However, the consumption of Ca, Mg, Se, and Cu in both e-waste and non-e-waste recyclers were lower than the RDA set by the WHO. Our findings are in line with similar studies in Malawi [69] and South Africa [70], where Ca and Se intake was lower among adult males. These previous findings suggest that micronutrient deficiency may be a common problem among males in sub-Saharan Africa. Between groups, the average Ca, Se, and Mg intake from the diet were lower in e-waste recyclers than non-e-waste recyclers. In contrast, the average Zn intake was lower in non-e-waste recyclers. Reasons for this pattern are not clear but may be attributed to poverty, job types, lack of access to a variety of micronutrient-rich foods, and perhaps lack of knowledge of optimal dietary practices. Studies have predicted that micronutrient (such as Ca, Cu, Mg, and Se) deficiencies may be associated with increased exposures to PM and heavy metals [30, 37, 71]. This suggests that, in populations such as informal sector e-waste recyclers, where exposures to PM and metals appear to be high, a public health strategy of increasing dietary consumption of micronutrients, including, if possible, taking supplements to help prevent the detrimental effects due to pollutant exposure is necessary.

### Relationship between dietary micronutrient intake and cardiovascular indices

This study found that dietary Ca intake was associated with reduced SBP and PP. This is consistent with other studies that examined dietary antioxidant intake and its relationship with BP [72–76]. However, in a double-blinded, placebo-controlled clinical trial, the intake of Ca-rich diet reduced DBP but not SBP [26, 77]. The reported differences in these studies may be due to variable physiologic-hormonal factors such as angiotensinogen and aldosterone that are known to regulate BP [78]. Thus, considering only environmental influences, in defining the role of Ca intake in regulating BP may be limiting. Furthermore, dietary Fe reduced SBP, PP, and AP levels in e-waste recyclers and non-e-waste recyclers. These are consistent with Lindberg et al. [79], who found that adequate Fe intake was associated with reduced SBP of adults. This reduction was also explicitly found among e-waste recyclers indicating that intake of Fe-rich diet may probably modify SBP levels. To the best of our knowledge, no previous data exist on dietary Fe intake and blood pressure of e-waste recyclers; therefore, inferences about causality may be premature.

Consistent with findings in other studies [80, 81], the unadjusted model revealed that Zn intake was associated with reduced SBP, indicating its deficiency as a risk factor for the occurrence of high BP. In contrast, other studies have reported that dietary intake of Zn does not affect BP in animals and humans. For example, in Taittonen et al. [82] study, dietary Zn was not linked with BP of healthy children in a 6-year prospective study. Similar findings were noted in animal studies where for four weeks, a Zn-deficient diet did not affect SBP or DBP in normotensive rats [83]. These inconsistent findings may be attributable to the degree of deficiency or adequacy of Zn intake, hypertensive status as well as the level of exposure to toxicants such as PM and heavy metals.

The exposure to high levels of PM_2.5_ coupled with Zn deficiency may perhaps impair the vascular nitric oxide (NO) system. This impairment may result in endothelial dysfunction and further reductions in endothelial-mediated vasoconstriction leading to increased BP levels [13, 14, 84]. Consequently, adequate intake of Zn-rich diets may be critical in maintaining endothelial cell integrity and BP, as Zn contains antioxidant and membrane-stabilizing properties [85–87]. More than half of e-waste and non-e-waste recyclers were found to be deficient in Zn. While the staple foods (e.g., groundnuts, millets, soybeans, and green leafy vegetables) consumed by these participants are Zn-rich, the patronage of western foods as well as urbanization, could have contributed to low intake of these traditional micronutrient-rich foods [88].

### Effects of dietary micronutrient intake on the association between PM_2.5_ exposure and BP outcomes

Results obtained in the current study generally provide evidence to support the hypothesis that intake of micronutrients-rich diets may modify the adverse effects of PM_2.5_ on BP, as reported by Schulz et al. [37]. For instance, in the joint effect model (Appendix 1D), adequate Fe intake lessened the effects of exposure to higher levels of PM_2.5_ on SBP after controlling for covariates in e-waste recyclers. These possible modifying effects of Fe intake may be attributed to the adequate consumption of Fe-rich diets assessed in e-waste as well as non-e-waste recyclers. Further studies such as experimental studies and clinical trials are, however, needed to confirm the effect of Fe intake on BP after exposure to PM. Our findings are consistent with Schulz et al. [37] and offer support for the assertion that the adverse effects of PM_2.5_ on BP may be reduced in participants who consume adequate amounts of micronutrient-rich diets.

Dietary Cu intake was also associated with increased PP of e-waste recyclers at higher PM_2.5_ exposure levels. Similar effects were observed when both groups were included in the regression model. Few studies have focused on the relationships between dietary Cu intake and BP levels. Results from an experimental study showed increases in BP in Cu-deficient rats [89]. In contrast, Lee et al. [90] found that dietary Cu intake significantly increased BP. These differences in findings suggest the need for further studies to better understand mechanisms of action in respect of Cu deficiency on BP indices, especially among toxicant exposed groups. Copper (Cu) is a significant component of antioxidant enzymes essential for the normal functions of the cardiovascular system [91]. Therefore, there is a possibility that deficiency of Cu coupled with high exposures to PM_2.5_ may lead to elevated BP and increased risks of cardiovascular events such as stroke. Furthermore, less than 20% of e-waste recyclers consumed adequate amounts of Cu though highly exposed to PM_2.5_, suggestive of the significantly high PP levels after PM_2.5_ exposure.

Antioxidants such as Mg, Se, Zn, Cu, and Zn inhibit oxidation reactions by reducing the number of free radicals produced and the level of harm they may cause [92, 93]. The intake of diets rich in such antioxidants may reduce the effects of reactive oxygen species (ROS) by removing their intermediates and terminating their chain reactions [94]. Nevertheless, e-waste recyclers who are highly exposed to PM_2.5,_ did not adequately consume these antioxidant-rich minerals. Although no significant relationships were observed, dietary micronutrients such as Mg, Se, Cu, and Zn intake similarly reduced adverse effects of PM_2.5_ on some BP measures. This finding suggests that adequate dietary intake of antioxidant-rich foods may subtly reduce the adverse effects of PM_2.5_ on BP.

Increasing evidence from experimental studies indicate that poor nutrition and pollutant exposure may interact and synergistically intensify the risk of cardiovascular diseases [95, 96]. Our results suggest that individuals who consumed adequate micronutrient-rich diets may have reduced adverse effects owing to the association between PM_2.5_ and BP. Several other studies have outlined the effects of adequate dietary micronutrient intake on cardiovascular health. As suggested in the findings by Schulz et al. [37], as well as the current study, adequate dietary micronutrient intake alone may not be sufficient to protect individuals against adverse effects of PM_2.5_ on BP. Steps that might reduce levels of PM_2.5_ exposure might include well-distributed PM monitoring networks in informal recycling e-waste sites. The establishment of health-based National Ambient Air Quality standards of PM_2.5_ and PM_10_ will significantly help control cardiovascular health effects in particularly exposed populations such as e-waste recyclers.

### Limitations and strengths

This study used a self-reported 2-day 24-h dietary recall in assessing micronutrient intake of participants from meals consumed. It is, therefore, liable for errors associated with the subjective measures. The memory-based dietary assessment method is largely pseudo-scientific, subject to recall bias, such as the underreporting of meal portions consumed. Given financial and logistical restraints, attempts were not made to evaluate biological indicators of oxidative stress, gene-environment interactions as well as participant’s sensitivity to oxidative stress that may probably influence micronutrient levels in the body [97–99]. Dietary micronutrient intake was individually assessed rather than collectively as part of a balanced diet. Also, we did not measure the physical activity levels of participants. Despite these limitations, the study had several unique strengths and contributions, as the impact of these limitations mentioned was probably offset by the more reliable and objective method used to measure PM_2.5_. We also believe that our study has value as the first to investigate the joint effects of PM_2.5_ and individual dietary micronutrient intake among e-waste recyclers in a natural setting. We measured BP and ambient measures of real-time personal air quality for almost two years. Also, computations of daily dietary micronutrient intake from whole foods rather than supplements were made.

## Conclusions

Consumption of Fe-rich foods was associated with a significant reduction in systolic BP, even at high PM exposure levels among e-waste recyclers. Therefore, environmental health promotion activities must factor dietary elements in health intervention programs to mitigate the effects of air pollution in highly polluted areas.

## Supplementary information

**Additional file 1. Appendix 1A**: Health Characteristics of e-waste and non-e-waste recyclers overtime. **Appendix 1B:** Relationship between E-waste exposure, Job Task and Daily Income Accrued on Micronutrient Intake: Random Effect Model. **Appendix 1C**: Association between PM_2.5_ Exposure and Blood Pressure Outcomes among E-waste and Non-E-waste Recyclers. **Appendix 1D**: Effects of Dietary Micronutrient intake on the relationship between PM_2.5_ and BP among e-waste recyclers. **Appendix 1E**: Graph of Dietary Micronutrient Intake of E-waste and Non-E-waste Recyclers Overtime.

## Data Availability

The datasets generated and/or analyzed during the current study are not publicly available due to privacy reasons, but are available from the corresponding author on reasonable request.

## Abbreviations

E-waste: Electronic waste
PM: Particulate matter
Ca: Calcium
Fe: Iron
Se: Selenium
Mg: Magnesium
Zn: Zinc
Cu: Copper
BP: Blood Pressure
SBP: Systolic Blood Pressure
DBP: Diastolic Blood Pressure
HR: Heart rate
PP: Pulse Pressure
ROS: Reactive Oxygen Species
BMI: Body Mass Index
MZ: Madina-Zongo
RDA: Recommended Daily Allowance
TSP: Total Suspended Particles
WHO: World Health Organization
USDA: United States Department of Agriculture
UNFPA: United Nations Population Funds

## References

[1] WHO (2019). Mortality and burden of disease from ambient air pollution-situation and trends.

[2] Chasant M (2019). Causes, effects and solutions for air pollution in Ghana.

[3] McAllister L. The human and environmental effects of e-waste. Popul Ref Bur. 2013.

[4] Jin L, Qiu J, Zhang Y, Qiu W, He X, Wang Y (2015). Ambient air pollution and congenital heart defects in Lanzhou, China. Environ Res Lett.

[5] Gangwar C, Choudhari R, Chauhan A, Kumar A, Singh A, Tripathi A (2019). Assessment of air pollution caused by illegal e-waste burning to evaluate the human health risk. Environ Int.

[6] Schultze B, Lind PM, Larsson A, Lind L (2014). Whole blood and serum concentrations of metals in a Swedish population-based sample. Scand J Clin Lab Invest.

[7] Ghorani-Azam A, Riahi-Zanjani B, Balali-Mood M. Effects of air pollution on human health and practical measures for prevention in Iran. J Res Med Sci. 2016;21. 10.4103/1735-1995.189646 PMC5122104 27904610

[8] WHO (1986). Early detection of occupational diseases.

[9] Rao X, Zhong J, Brook RD, Rajagopalan S (2018). Effect of particulate matter air pollution on cardiovascular oxidative stress pathways. Antioxid Redox Signal.

[10] Genc S, Zadeoglulari Z, Fuss SH, Genc K. The adverse effects of air pollution on the nervous system. J Toxicol. 2012;2012. 10.1155/2012/782462 PMC3317189 22523490

[11] Shukla A, Bunkar N, Kumar R, Bhargava A, Tiwari R, Chaudhury K (2018). Air pollution associated epigenetic modifications: transgenerational inheritance and underlying molecular mechanisms. Sci Total Environ.

[12] Wright JC, Ding Y (2016). Pathophysiological effects of particulate matter air pollution on the central nervous system. Environ Dis.

[13] Xia B, Zhou Y, Zhu Q, Zhao Y, Wang Y, Ge W (2019). Personal exposure to PM2. 5 constituents associated with gestational blood pressure and endothelial dysfunction. Environ Pollut.

[14] Dai J, Sun C, Yao Z, Chen W, Yu L, Long M (2016). Exposure to concentrated ambient fine particulate matter disrupts vascular endothelial cell barrier function via the IL-6/HIF-1α signaling pathway. FEBS Open Bio.

[15] Hennig PMC, Gamble MV, Surh Y-J, Kresty LA, Frank N (2018). The role of nutrition in influencing mechanisms involved in environmentally mediated diseases. Rev Environ Health.

[16] Petriello MC, Newsome BJ, Dziubla TD, Hilt JZ, Bhattacharyya D, Hennig B (2014). Modulation of persistent organic pollutant toxicity through nutritional intervention: emerging opportunities in biomedicine and environmental remediation. Sci Total Environ.

[17] Petriello MC, Newsome B, Hennig B (2014). Influence of nutrition in PCB-induced vascular inflammation. Environ Sci Pollut Res.

[18] Whyand T, Hurst J, Beckles M, Caplin M (2018). Pollution and respiratory disease: can diet or supplements help? A review. Respir Res.

[19] Liu Z, Ren Z, Zhang J, Chuang C-C, Kandaswamy E, Zhou T (2018). Role of ROS and nutritional antioxidants in human diseases. Front Physiol.

[20] Hoffman, Hennig B (2017). Protective influence of healthful nutrition on mechanisms of environmental pollutant toxicity and disease risks. Ann N Y Acad Sci.

[21] Hennig B, Ormsbee L, McClain CJ, Watkins BA, Blumberg B, Bachas LG (2012). Nutrition can modulate the toxicity of environmental pollutants: implications in risk assessment and human health. Environ Health Perspect.

[22] Rosique-Esteban N, Guasch-Ferré M, Hernández-Alonso P, Salas-Salvadó J (2018). Dietary magnesium and cardiovascular disease: A review with emphasis in epidemiological studies. Nutrients..

[23] DiNicolantonio, Liu J, O’Keefe JH. Magnesium for the prevention and treatment of cardiovascular disease. Arch Dis Child. 2018. 10.1136/openhrt-2018-000775 PMC6045762 30018772

[24] Cunha AR, Umbelino B, Correia ML, Neves MF. Magnesium and vascular changes in hypertension. Int J Hypertens. 2012;2012. 10.1155/2012/754250 PMC3299255 22518291

[25] Cormick G, Ciapponi A, Cafferata ML, Belizán JM. Calcium supplementation for prevention of primary hypertension. Cochrane Database Syst Rev. 2015;(6). 10.1002/14651858.CD010037.pub2 PMC6486289 26126003

[26] Entezari MH. The effect of supplementary calcium on blood pressure in healthy adult women aged 18–30 years in Tehran, Iran. J Educ Health Promot. 2015;4. 10.4103/2277-9531.162388 PMC4579758 26430694

[27] Tang, Wang D-G, Li J, Li X-H, Wang Q, Liu N, et al. Relationships between micronutrient losses in sweat and blood pressure among heat-exposed steelworkers. Ind Health. 2016:2014–0225. 10.2486/indhealth.2014-0225 PMC4939859 27087421

[28] Possamai FP, Júnior SÁ, Parisotto EB, Moratelli AM, Inácio DB, Garlet TR (2010). Antioxidant intervention compensates oxidative stress in blood of subjects exposed to emissions from a coal electric-power plant in South Brazil. Environ Toxicol Pharmacol.

[29] Limón-Pacheco J, Gonsebatt ME (2009). The role of antioxidants and antioxidant-related enzymes in protective responses to environmentally induced oxidative stress. Mutat Res Genet Toxicol Environ Mutagen.

[30] Miller CN, Rayalam S (2017). The role of micronutrients in the response to ambient air pollutants: potential mechanisms and suggestions for research design. J Toxicol Environ Health B.

[31] Ekpenyong CE (2017). Micronutrient vitamin deficiencies and cardiovascular disease risk: advancing current understanding. Eur J Prev Med.

[32] McKeag NA, McKinley MC, Woodside JV, Harbinson MT, McKeown PP (2012). The role of micronutrients in heart failure. J Acad Nutr Diet.

[33] DiNicolantonio, O’Keefe JH, Wilson W (2018). Subclinical magnesium deficiency: a principal driver of cardiovascular disease and a public health crisis. Open Heart.

[34] Porpora MG, Piacenti I, Scaramuzzino S, Masciullo L, Rech F, Benedetti PP (2019). Environmental contaminants exposure and preterm birth: a systematic review. Toxics..

[35] Lanphear BP (2015). The impact of toxins on the developing brain. Annu Rev Public Health.

[36] Balbus JM, Boxall AB, Fenske RA, McKone TE, Zeise L (2013). Implications of global climate change for the assessment and management of human health risks of chemicals in the natural environment. Environ Toxicol Chem.

[37] Schulz AJ, Mentz GB, Sampson NR, Dvonch JT, Reyes AG, Izumi B (2015). Effects of particulate matter and antioxidant dietary intake on blood pressure. Am J Public Health.

[38] Izumi BT, Zenk SN, Schulz AJ, Mentz GB, Wilson C (2011). Associations between neighborhood availability and individual consumption of dark-green and orange vegetables among ethnically diverse adults in Detroit. J Am Diet Assoc.

[39] Amoabeng Nti AA, Arko-Mensah J, Botwe PK, Dwomoh D, Kwarteng L, Takyi SA (2020). Effect of particulate matter exposure on respiratory health of e-waste workers at agbogbloshie, Accra, Ghana. Int J Environ Res Public Health.

[40] Feldt T, Fobil JN, Wittsiepe J, Wilhelm M, Till H, Zoufaly A (2014). High levels of PAH-metabolites in urine of e-waste recycling workers from Agbogbloshie, Ghana. Sci Total Environ.

[41] Wittsiepe J, Feldt T, Till H, Burchard G, Wilhelm M, Fobil JN (2017). Pilot study on the internal exposure to heavy metals of informal-level electronic waste workers in Agbogbloshie, Accra, Ghana. Environ Sci Pollut Res.

[42] Asampong E, Dwuma-Badu K, Stephens J, Srigboh R, Neitzel R, Basu N (2015). Health seeking behaviours among electronic waste workers in Ghana. BMC Public Health.

[43] Srigboh RK, Basu N, Stephens J, Asampong E, Perkins M, Neitzel RL (2016). Multiple elemental exposures amongst workers at the Agbogbloshie electronic waste (e-waste) site in Ghana. Chemosphere..

[44] Simon S (2018). From Europe, to the Agbogbloshie scrapyard.

[45] United Nations Population Fund (2018). Reaching the underserved: UNFPA Youth Fellows Organizes Outreach at Old Fadama.

[46] Amoyaw-Osei Y, Agyekum OO, Pwamang JA, Mueller E, Fasko R, Schluep M (2011). Ghana e-waste country assessment. SBC e-waste Afr Project.

[47] Laskaris Z, Milando C, Batterman S, Mukherjee B, Basu N, O'Neill MS, et al. Derivation of time-activity data using wearable cameras and measures of personal inhalation exposure among workers at an informal electronic-waste recovery site in Ghana. Ann Work Expo Health. 2019. 10.1093/annweh/wxz056 PMC6788341 31334545

[48] Alkhajah TA, Reeves MM, Eakin EG, Winkler EA, Owen N, Healy GN (2012). Sit–stand workstations: a pilot intervention to reduce office sitting time. Am J Prev Med.

[49] Zeba AN, Delisle HF, Renier G. Dietary patterns and physical inactivity, two contributing factors to the double burden of malnutrition among adults in Burkina Faso, West Africa. J Nutr Sci. 2014;3. 10.1017/jns.2014.11 PMC4473138 26101618

[50] Boateng GP (2014). The development of a photographic food atlas with portion sizes of commonly consumed carbohydrate foods in Accra.

[51] Padwal R, Polley G, McLean D, Thompson A, Morales F, Ringrose J (2017). [PP. 16.08] an assessment of the accuracy of home blood pressure monitors when used in device owners. J Hypertens.

[52] Cao X, Song C, Guo L, Yang J, Deng S, Xu Y, et al. Quality control and validation of oscillometric blood pressure measurements taken during an epidemiological investigation. Medicine. 2015;94(37). 10.1097/MD.0000000000001475 PMC4635802 26376388

[53] Zheng M, Xu X, Wang X, Huo Y, Xu X, Qin X, et al. Age, arterial stiffness, and components of blood pressure in Chinese adults. Medicine. 2014;93(29). 10.1097/MD.0000000000000262 PMC4602627 25546666

[54] Sesso HD, Stampfer MJ, Rosner B, Hennekens CH, Gaziano JM, Manson JE (2000). Systolic and diastolic blood pressure, pulse pressure, and mean arterial pressure as predictors of cardiovascular disease risk in men. Hypertension..

[55] Chow JC, Watson JG (1998). Guideline on speciated particulate monitoring.

[56] Widman S (2011). Comparison of PM 2.5 Samplers in residential environments.

[57] Mahan LK, Raymond JL (2016). Krause’s food & the nutrition care process.

[58] For EPOIG, Children RRI (2011). Expert panel on integrated guidelines for cardiovascular health and risk reduction in children and adolescents: summary report. Pediatrics..

[59] Textor J, Hardt J, Knüppel S (2011). DAGitty: a graphical tool for analyzing causal diagrams. Epidemiology..

[60] Ahad NA, Yin TS, Othman AR, Yaacob CR (2011). Sensitivity of normality tests to non-normal data. Sains Malaysiana.

[61] Nachvak SM, Moradi S, Mostafai R, Sharafi K (2016). The role of nutrition in reducing the harmful effects of dust on human health: a review study.

[62] Majkova Z, Toborek M, Hennig B (2010). The role of caveolae in endothelial cell dysfunction with a focus on nutrition and environmental toxicants. J Cell Mol Med.

[63] Breitner S, Peters A, Zareba W, Hampel R, Oakes D, Wiltshire J (2019). Ambient and controlled exposures to particulate air pollution and acute changes in heart rate variability and repolarization. Sci Rep.

[64] Xie X, Wang Y, Yang Y, Xu J, Zhang Y, Tang W (2018). Long-term exposure to fine particulate matter and tachycardia and heart rate: results from 10 million reproductive-age adults in China. Environ Pollut.

[65] Cole-Hunter T, de Nazelle A, Donaire-Gonzalez D, Kubesch N, Carrasco-Turigas G, Matt F (2018). Estimated effects of air pollution and space-time-activity on cardiopulmonary outcomes in healthy adults: a repeated measures study. Environ Int.

[66] Dong W, Pan L, Li H, Miller M, Loh M, Wu S (2018). Association of size-fractionated indoor particulate matter and black carbon with heart rate variability in healthy elderly women in Beijing. Indoor Air.

[67] UNDP (2018). Northern Ghana Human Development Report 2018. Bridging the poverty gap and fostering socio-economic transformation and empowerment to contribute to human development for all.

[68] Twinamasiko B, Lukenge E, Nabawanga S, Nansalire W, Kobusingye L, Ruzaaza G, Bajunirwe F. Sedentary lifestyle and hypertension in a periurban area of Mbarara, South western Uganda: a population based cross sectional survey. Int J Hypertens. 2018;2018. 10.1155/2018/8253948 PMC5960550 29854432

[69] Joy EJ, Kumssa DB, Broadley MR, Watts MJ, Young SD, Chilimba AD (2015). Dietary mineral supplies in Malawi: spatial and socioeconomic assessment. BMC Nutr.

[70] Kolahdooz F, Spearing K, Sharma S (2013). Dietary adequacies among South African adults in rural KwaZulu-Natal. PLoS One.

[71] Bharatraj DK, Yathapu SR (2018). Nutrition-pollution interaction: an emerging research area. Indian J Med Res.

[72] Silva N, Araújo S (2017). Mineral intake and blood pressure control of Brazilian elderly. MOJ Gerontol Ger.

[73] Cormick G, Belizán JM (2019). Calcium intake and health. Nutrients..

[74] Khanam F, Hossain B, Mistry SK, Mitra DK, Raza WA, Rifat M (2018). The association between daily 500 mg calcium supplementation and lower pregnancy-induced hypertension risk in Bangladesh. BMC Pregnancy Childbirth.

[75] Villa-Etchegoyen C, Lombarte M, Matamoros N, Belizán JM, Cormick G (2019). Mechanisms involved in the relationship between low calcium intake and high blood pressure. Nutrients..

[76] Kim BSY, Choi M-K (2012). Daily calcium intake and its relation to blood pressure, blood lipids, and oxidative stress biomarkers in hypertensive and normotensive subjects. Nutr Res Pract.

[77] Drouin-Chartier J-P, Gigleux I, Tremblay AJ, Poirier L, Lamarche B, Couture P (2014). Impact of dairy consumption on essential hypertension: a clinical study. Nutr J.

[78] Vaidya A, Brown JM, Williams JS (2015). The renin–angiotensin–aldosterone system and calcium-regulatory hormones. J Hum Hypertens.

[79] Lindberg J, Norman M, Westrup B, Domellöf M, Berglund SK (2017). Lower systolic blood pressure at age 7 y in low-birth-weight children who received iron supplements in infancy: results from a randomized controlled trial. Am J Clin Nutr.

[80] Kim (2013). Dietary zinc intake is inversely associated with systolic blood pressure in young obese women. Nutr Res Pract.

[81] Wang Y, Jia X-F, Zhang B, Wang Z-H, Zhang J-G, Huang F-F (2018). Dietary zinc intake and its association with metabolic syndrome indicators among Chinese adults: an analysis of the China Nutritional Transition Cohort Survey 2015. Nutrients..

[82] Taittonen L, Nuutinen M, Räsänen L, Mussalo-Rauhamaa H, Turtinen J, Uhari M (1997). Lack of association between copper, zinc, selenium and blood pressure among healthy children. J Hum Hypertens.

[83] Sato M, Kurihara N, Moridaira K, Sakamoto H, Tamura JI, Wada O (2003). Dietary Zn deficiency does not influence systemic blood pressure and vascular nitric oxide signaling in normotensive rats. Biol Trace Elem Res.

[84] Daiber A, Xia N, Steven S, Oelze M, Hanf A, Kröller-Schön S (2019). New therapeutic implications of endothelial nitric oxide synthase (eNOS) function/dysfunction in cardiovascular disease. Int J Mol Sci.

[85] Rainsford KD, Milanino R, Sorenson J, Velo G (1998). Copper and zinc in inflammatory and degenerative diseases.

[86] Skene K, Walsh SK, Okafor O, Godsman N, Barrows C, Meier P (2019). Acute dietary zinc deficiency in rats exacerbates myocardial ischaemia–reperfusion injury through depletion of glutathione. Br J Nutr.

[87] Daiber A, Steven S, Weber A, Shuvaev VV, Muzykantov VR, Laher I (2017). Targeting vascular (endothelial) dysfunction. Br J Pharmacol.

[88] de Jager I, Giller KE, Brouwer ID (2018). Food and nutrient gaps in rural Northern Ghana: Does production of smallholder farming households support adoption of food-based dietary guidelines?. PLoS One.

[89] Klevay LM, Halas ES (1991). The effects of dietary copper deficiency and psychological stress on blood pressure in rats. Physiol Behav.

[90] Lee LE-S, Oh S-Y, Park H-R, Ro H-K, Heo Y-R (2015). Daily copper and manganese intakes and their relation to blood pressure in normotensive adults. Clin Nutr Res.

[91] Kurutas EB (2015). The importance of antioxidants which play the role in cellular response against oxidative/nitrosative stress: current state. Nutr J.

[92] Lee SR. Critical role of zinc as either an antioxidant or a prooxidant in cellular systems. Oxidative Med Cell Longev. 2018;2018. 10.1155/2018/9156285 PMC5884210 29743987

[93] Mehta SK, Gowder SJ. Members of antioxidant machinery and their functions. Basic Princ Clin Significance Oxid Stress. 2015:59–85.

[94] Tan BL, Norhaizan ME, Liew WP, Sulaiman Rahman H. Antioxidant and oxidative stress: a mutual interplay in age-related diseases. Front Pharmacol. 2018;9:1162. 10.3389/fphar.2018.01162 PMC6204759 30405405

[95] Péter S, Holguin F, Wood LG, Clougherty JE, Raederstorff D, Antal M (2015). Nutritional solutions to reduce risks of negative health impacts of air pollution. Nutrients..

[96] Lorzadeh E, Salehi-Abargouei A (2017). How nutrition might modify the possible effects of air pollution on cardiovascular diseases’ risk?. J Environ Health Sustain Dev.

[97] González J, Valls N, Brito R, Rodrigo R (2014). Essential hypertension and oxidative stress: New insights. World J Cardiol.

[98] Narasimha Rai K, Kumari NS, Damodara Gowda K, Swathi K (2013). The evaluation of micronutrients and oxidative stress and their relationship with the lipid profile in healthy adults. J Clin Diagn Res.

[99] Minelli C, Wei I, Sagoo G, Jarvis D, Shaheen S, Burney P (2011). Interactive effects of antioxidant genes and air pollution on respiratory function and airway disease: a HuGE review. Am J Epidemiol.

