# Supplementary material for: Micronutrient-rich dietary intake is associated with a reduction in the effects of particulate matter on blood pressure among electronic waste recyclers at Agbogbloshie, Ghana
Source: BMC Public Health. 2020 Jul 6;20:1067. doi: 10.1186/s12889-020-09173-8 (PMC7339459; doi:10.1186/s12889-020-09173-8)
Supplement: Supplementary file 1 — Additional file 1. Appendix 1A: Health Characteristics of e-waste and non-e-waste recyclers overtime. Appendix 1B: Relationship between E-waste exposure, Job Task and Daily Income Accrued on Micronutrient Intake: Random Effect Model. Appendix 1C: Association between PM2.5 Exposure and Blood Pressure Outcomes among E-waste and Non-E-waste Recyclers. Appendix 1D: Effects of Dietary Micronutrient intake on the relationship between PM2.5 and BP among e-waste recyclers. Appendix 1E: Graph of Dietary Micronutrient Intake of E-waste and Non-E-waste Recyclers Overtime. [file 12889_2020_9173_MOESM1_ESM.doc]

**SUPPLEMENTARY DATA**

**APPENDIXES**

**Appendix 1A**: **Health Characteristics of e-waste and non-e-waste recyclers overtime.**

| Variables | **E-waste Recyclers** | | | | | **Non-E-waste Recyclers** | | | | |
| --- | --- | --- | --- | --- | --- | --- | --- | --- | --- | --- |
|  | Baseline | Midline | End line | df | F | Baseline | Midline | End-line | df | F |
| Systolic BP | 123.06±12.36 | 119.85±10.75 | 119.62±14.12 | 2 | **3.56*** | 128.57±17.13 | 127.00±17.30 | 129.13±20.07 | 2 | 0.44 |
| Diastolic BP | 73.61±9.90 | 72.19±8.48 | 70.90±10.91 | 2 | 2.41 | 76.46±12.12 | 75.52±12.55 | 75.96±13.18 | 2 | 0.51 |
| Heart Rate | 73.69±11.50 | 73.44±9.97 | 73.82±10.74 | 2 | 0.08 | 74.38±13.21 | 69.36±10.86 | 73.62±12.93 | 2 | **3.39*** |
| Pulse Pressure | 49.46±11.23 | 47.67±11.22 | 48.71±12.17 | 2 | 1.56 | 52.11±9.97 | 51.48±10.80 | 53.18±10.89 | 2 | 0.17 |
| Arterial Pressure | 90.09±9.39 | 88.07±7.65 | 87.14±10.62 | 2 | **3.25*** | 93.83±13.17 | 92.68±13.38 | 93.68±14.96 | 2 | 0.54 |
| PM_2.5_ | 80.39±62.95 | 70.49±24.32 | 70.49±31.51 | 2 | 1.74 | 39.03±17.95 | 49.50±49.59 | 87.50±84.92 | 2 | **4.24*** |
| Body Mass Index (BMI) | 22.52±2.94 | 23.96±3.02 | 24.18±3.40 | 2 | **20.41***** | 23.82±3.33 | 24.14±3.52 | 24.11±3.84 | 2 | 0.76 |

p-values notations: ***p<0.001 **p<0.01 *p<0.05

**Appendix 1B:** **Relationship between E-waste exposure, Job Task and Daily Income Accrued on Micronutrient Intake: Random Effect Model**

| **Variables** | **Calcium (Ca)**  **β (95% CI)** | **Iron (Fe)**  **β (95% CI)** | **Copper (Cu)**  **β (95% CI)** | **Se (Selenium)**  **β (95% CI)** | **Magnesium (Mg)**  **β (95% CI)** | **Zinc (Zn)**  **β (95% CI)** |
| --- | --- | --- | --- | --- | --- | --- |
| **Location** |  |  |  |  |  |  |
| Madina Zongo | 1 |  |  |  |  |  |
| E-waste | -0.05 (-0.20, 0.10) | **0.15*** (0.02, 0.29) | 0.09(-0.13, 0.30) | -0.25(-0.58, 0.07) | **-0.43****(-0.69, -0.17) | **0.19****(0.06, 0.31) |
| **Daily Income** |  |  |  |  |  |  |
| < GH ¢ 20 | 1 |  |  |  |  |  |
| GH ¢ 20-100 | 0.10 (-0.03, 0.24) | 0.08(-0.04, 0.21) | 0.05(-0.15, 0.24) | 0.05(-0.24, 0.35) | 0.07(-0.17, 0.31) | **0.13***(0.02, 0.24) |
| GH ¢ 101-200 | 0.18(-0.03, 0.39) | 0.11 (-0.08, 0.30) | 0.12(-0.17, 0.43) | 0.08(-0.37, 0.53) | 0.11(-0.26, 0.48) | **0.23****(0.06, 0.40) |
| >GH ¢200 | **0.24*** (0.03, 0.45) | **0.24***(0.05, 0.44) | 0.17(-0.14, 0.47) | 0.10(-0.37, 0.58) | 0.01(-0.37, 0.38) | **0.21***(0.04, 0.38) |
| **Job Type** |  |  |  |  |  |  |
| Burner | 1 |  |  |  |  |  |
| Dismantler | 0.06 (-0.09, 0.20) | 0.11(-0.02, 0.24) | 0.09 (-0.11, 0.30) | 0.27(-0.04, 0.59) | 0.11(-0.14, 0.36) | 0.03(-0.09, 0.14) |
| Sorter | 0.07 (-0.20, 0.35) | 0.15(-0.10, 0.40) | 0.03 (-0.36, 0.43) | 0.01(-0.58, 0.60) | -0.08(-0.55, 0.39) | 0.06(0.16, 0.28) |
| Collector | 0.07 (-0.13, 0.28) | 0.08(-0.10, 0.27) | 0.15(-0.14, 0.45) | **0.47***(0.02, 0.92) | 0.27(-0.10, 0.63) | 0.10(-0.07, 0.26) |

p-values notations: ***p<0.001 **p<0.01 *p<0.05

**Appendix 1C: Association between PM_2.5_ Exposure and Blood Pressure Outcomes among E-waste and Non-E-waste Recyclers**

| **BP outcomes** | **E-waste Recyclers**  **β [95%CI:]** | **Non-E-waste Recyclers**  **β [95%CI:]** |
| --- | --- | --- |
| Systolic BP | -0.019[-0.046, 0.007] | -0.036[-0.078, 0.005] |
| Diastolic BP | -0.007[-0.044, 0.030] | -0.048[-0.096, -0.0002] |
| Heart Rate | **0.061***[0.007, 0.116] | -0.007[-0.066, 0.051] |
| Pulse Pressure | -0.038[-0.103, 0.027] | -0.017[-0.081, 0.047] |
| Arterial Pressure | -0.013[-0.041, 0.015] | **-0.043***[-0.085, -0.001] |

Model adjusted for age, smoking status, total calories consumed, dietary diversity scores and BMI

P-value notations: *p<0.05

| **Variables** | **Systolic Blood Pressure (SBP)** | **Diastolic Blood Pressure (DBP)** | **Pulse Pressure (PP)** | **Arterial Pressure (AP)** | **Heart Rate (HR)** |
| --- | --- | --- | --- | --- | --- |
|  | **β [95% CI]** | **β [95% CI]** | **β [95% CI]** | **β [95% CI]** | **β [95% CI]** |
| PM_2.5_ | **-**0.021 [-0.048, 0.005] | -0.010 [-0.046, 0.027] | -0.038 [-0.1022, -0.025] | -0.015 [-0.043, 0.013] | 0.046 [-0.008, 0.099] |
| Ca | -0.017 [-0.049, 0.015] | 0.005 [-0.038, 0.049] | -0.046 [-0.122, 0.031] | -0.005 [-0.039, 0.028] | -0.045 [-0.112, 0.018] |
|  |  |  |  |  |  |
| PM_2.5_ | -0.017 [-0.044, 0.011] | -0.011 [-0.047, 0.026] | -0.032 [-0.097, -0.033] | -0.014 [-0.043, 0.014] | 0.048 [-0.005, 0.103] |
| Mg | 0.003 [-0.011, 0.018] | 0.012 [-0.008, 0.032] | -0.010 [-0.046, 0.025] | 0.008 [-0.007, 0.023] | 0.025 [0.004, 0.054] |
|  |  |  |  |  |  |
| PM_2.5_ | -0.020 [-0.046, 0.008] | -0.010 [-0.046, 0.030] | -0.036 [-0.099, 0.028] | -0.015 [-0.042, 0.013] | 0.027 [-0.010, 0.064] |
| Fe | **-0.040*** [-0.075, -0.005] | -0.029 [-0.076, 0.019] | -0.045 [-0.129, 0.039] | -0.034 [-0.070, 0.003] | -0.029 [-0.086, 0.028] |
|  |  |  |  |  |  |
| PM_2.5_ | -0.025 [-0.074, 0.024] | -0.012 [-0.050, 0.026] | -0.036 [-0.082, 0.030] | -0.016 [-0.045, 0.013] | 0.051 [-0.005, 0.106] |
| Se | 0.030 [0.004, 0.056] | -0.008 [-0.026, 0.010] | 0.013 [-0.018, 0.045] | -0.004 [-0.018, 0.010] | -0.006 [-0.032, 0.021] |
|  |  |  |  |  |  |
| PM_2.5_ | -0.020 [-0.047, -0.006] | -0.010 [-0.046, 0.026] | -0.045 [-0.100, 0.027] | -0.015 [-0.042, 0.013] | 0.049 [-0.005, 0.103] |
| Cu | 0.016 [-0.002, 0.035] | -0.004 [-0.029, 0.021] | **0.044*** [0.001, 0.088] | 0.006 [-0.013, 0.025] | -0.016 [-0.052, 0.021] |
|  |  |  |  |  |  |
| PM_2.5_ | -0.019 [-0.046, -0.007] | -0.009 [-0.045, 0.028] | -0.036 [-0.100, 0.028] | -0.014 [-0.041, 0.014] | 0.048 [0.011, 0.102] |
| Zn | -0.018 [-0.063, 0.026] | -0.041 [-0.102, 0.020] | 0.012 [-0.095, 0.121] | -0.030 [-0.077, 0.016] | -0.005 [-0.085, 0.096] |

**Appendix 1D: Effects of Dietary Micronutrient intake on the relationship between PM_2.5_ and BP among e-waste recyclers**

Random effects adjustment was made for age, income, BMI, smoking status, marital status, biomass exposure, total calories consumed and dietary diversity scores in the model. p<0.025*, for which reason their values were boldened.

**APPENDIX 1E: Graph of Dietary Micronutrient Intake of E-waste and Non-E-waste Recyclers Overtime**

Graph of Dietary Micronutrient Intake of E-waste and Non-E-waste Recyclers Overtime
